# Utility of *Clostridium difficile* Toxin B for Inducing Anti-Tumor Immunity

**DOI:** 10.1371/journal.pone.0110826

**Published:** 2014-10-23

**Authors:** Tuxiong Huang, Shan Li, Guangchao Li, Yuan Tian, Haiying Wang, Lianfa Shi, Gregorio Perez-Cordon, Li Mao, Xiaoning Wang, Jufang Wang, Hanping Feng

**Affiliations:** 1 School of Bioscience and Bioengineering, South China University of Technology (SCUT), Guangzhou, China; 2 Department of Microbial Pathogenesis, University of Maryland Dental School, Baltimore, Maryland, United States of America; 3 Department of Oncology and Diagnostics, University of Maryland Dental School, Baltimore, Maryland, United States of America; 4 Institute of Life Science, General Hospital of the People’s Liberation Army, Beijing, China; Institute Pasteur, France

## Abstract

*Clostridium difficile* toxin B (TcdB) is a key virulence factor of bacterium and induces intestinal inflammatory disease. Because of its potent cytotoxic and proinflammatory activities, we investigated the utility of TcdB in developing anti-tumor immunity. TcdB induced cell death in mouse colorectal cancer CT26 cells, and the intoxicated cells stimulated the activation of mouse bone marrow-derived dendritic cells and subsequent T cell activation *in vitro*. Immunization of BALB/c mice with toxin-treated CT26 cells elicited potent anti-tumor immunity that protected mice from a lethal challenge of the same tumor cells and rejected pre-injected tumors. The anti-tumor immunity generated was cell-mediated, long-term, and tumor-specific. Further experiments demonstrated that the intact cell bodies were important for the immunogenicity since lysing the toxin-treated tumor cells reduced their ability to induce antitumor immunity. Finally, we showed that TcdB is able to induce potent anti-tumor immunity in B16-F10 melanoma model. Taken together, these data demonstrate the utility of *C. difficile* toxin B for developing anti-tumor immunity.

## Introduction

TcdB is one of key virulence factors of *Clostridium difficile* (*C. difficile*), a principal cause of antibiotic-associated diarrhea and pseudomembranous colitis [1]. This toxin is a single-chain protein consisting of several functional domains including those for receptor binding, delivery, and effector glucosyltransferase activity [2], [3], [4]. Once inside the host cell cytoplasm, the glucosyltransferase domain of the toxin can glucosylate small Rho GTPase family proteins, such as RhoA, CDC42, and Rac1, causing disruption of the cytoskeleton and interfering with other signaling pathways [5], [6], [7]. TcdB is highly cytotoxic for many cell lines [8], [9], [10], killing cells by inducing apoptosis [11], [12], [13] or necrosis [14], [15]. Importantly, TcdB is also proinflammatory, capable of inducing the production of cytokines and chemokines in target cells [16], [17], [18] and causing inflammatory disease, such as pseudomembranous colitis, in the host [10], [19]. In the process of inflammation, antigen presenting cells such as monocytes and dendritic cells may be activated [20]. In addition to playing important roles in inflammation, macrophages and dendritic cells are critical in regulating the innate immune response and inducing adaptive immunity [21].

The induction of immunogenic tumor-cell death can be very useful in the application of cancer therapy, since this way may elicit memory anti-tumor immunity, protecting host against chemotherapy-resistant cancer cells and cancer stem cells [22], [23]. We have previously found that tumor cells undergoing apoptosis in a stressful and/or inflammatory microenvironment are highly immunogenic, capable of activating dendritic cells and eliciting tumor-specific immunity [24], [25], [26]. Recent studies found that certain chemotherapeutic drugs, such as anthracyclines [27], [28] and ionizing irradiation [29], can induce tumor cells to undergo immunogenic cell death and stimulate antitumor immunity *in vivo*. However, few reports show that bacterial toxins induce immunogenic death of cancer cells [30], [31].

Since TcdB possesses potent cytotoxic and pro-inflammatory activities, we hypothesized that this toxin is capable of inducing antitumor immunity. In this study, we found that TcdB-intoxicated tumor cells are highly immunogenic and capable of inducing potent, long-term, and specific anti-tumor immunity. Our data demonstrate that this bacterial toxin may be utilized to induce antitumor immunity, thus provide insight into the utility of *C. difficile* toxins for designing effective anti-tumor vaccines and immunotherapies against cancers.

## Materials and Methods

### Ethics statement

All animals were handled and cared for according to China Animal Care and Use Committee guidelines or Institutional Animal Care and Use Committee guidelines and in accordance with the recommendations in the Guide for the Care and Use of Laboratory Animals of the National Institutes of Health. The protocols were approved by the Committee on the Ethics of Animal Experiments of the Tufts University Cummings School of Veterinary Medicine (Protocol #2008-GR20) or at University of Maryland School of Medicine (Protocol #D120301).

### Mice, cell lines, and toxins

Six- to 10-week-old male BALB/c or C57BL/6 mice were purchased from the Medical Experimental Animal Center (Guangdong, China) and Jackson Laboratory. All mice used in the experiments were housed in groups of 5 per cage under the same conditions. Food, water, bedding, and cages were autoclaved. Murine colon adenocarcinoma cell lines CT26 and CT26.CL25 (CT26 cells expressing the model antigen β-galactosidase) [34], the myeloma cell line p3x63Ag8.653, and the melanocytoma cell line B16-F10 were obtained from the American Type Culture Collection (ATCC, Manassas, VA, USA). Cells were maintained in Dulbecco’s modified Eagle medium (DMEM; Invitrogen, Carlsbad, CA, USA) containing 10% fetal bovine serum (Invitrogen), 100 U/ml penicillin, 100 µg/ml streptomycin (Invitrogen), 2 mM L-glutamine (Invitrogen), and 1 mM pyruvate acid (Invitrogen). Full-length recombinant TcdB were purified from total crude extract of *Bacillus megaterium* as described previously [38]. The biological activity of recombinant TcdB is essentially identical to native toxin [38]. The highly purified recombinant TcdB that appeared as a single band on SDS-PAGE, and was absent of detectable TLR2 (Toll like receptor 2) and TLR4 ligand activity as determined by bioassays [38], [39], was used in this study.

### Cytotoxicity assays

Cells were exposed to 500 ng/ml of TcdB for different time, and then harvested and stained with 1 µg/ml of propidium iodide (PI) for 15 minutes. The percentage of PI positive cells was analyzed by flow cytometry using FACS Calibur and CellQuest software (BD Biosciences, Mountain View, CA, USA).

### Stimulation of T cells by tumor loaded DCs *in vitro*

BALB/c mouse bone marrow dendritic cells (BMDCs) were generated as described previously [25], [26]. More than 90% of these cells were CD11c^+^ DCs [43]. BMDCs were pulsed with live or TcdB-treated CT26 cells in 24-well plates at a ratio of 1∶1 overnight. Tumor-loaded DCs or unpulsed DCs (matured by LPS) were co-cultured in 24-well plates with autologous splenocytes at a ratio of 1∶30. After 1 week, splenocytes were restimulated using the same antigen presenting cells (APCs) as the initial stimulation. Mouse recombinant IL-2 (50 IU/ml; Invitrogen) was added on days 2 and 7 of culture. Seven days post the second stimulation the supernatants of the cultures were collected for measuring IFN-γ production by ELISA.

### Mouse immunization and challenge

CT26 cells were exposed to 500 ng/ml of TcdB for 6 h and then washed 3 times in PBS. Six hours of toxin treatment on CT26 cells did not result in necrosis of the cells as measured by trypan blue staining. To generate lysate, the toxin-exposed (6 h) cells were freeze/thawed for 5 cycles. For the prophylactic model, groups of 6-week old BALB/c mice (5 to 8 mice per group) were injected with PBS or 10^6^ cells/mouse of TcdB-treated CT26 cells, or toxin-treated CT26 lysate subcutaneously (SC) into the right groin twice on days −14 and −7. A total of 10^5^ (LD_100_) viable CT26 cells, determined by trypan blue exclusion, were inoculated SC into the right flank of the mice on day 0. For the therapeutic model, mice were challenged with CT26 cells 4 h prior to immunization. In some experiments, groups of mice were challenged with 5×10^5^ (LD_100_) live myeloma p3x63Ag8.653 cells 4 h before injection with PBS or 10^6^ toxin-treated CT26 cells to assess the specificity of anti-tumor immunity. In rechallenge experiments, 10^6^ of live CT26 cells (10-fold LD_100_) were injected into the left flank of surviving mice 3 months after the first challenge. For the mouse melanoma cancer model, the B16-F10 tumor cells were treated with 500 ng/ml of TcdB for 6 h before extensive washing. PBS or 2×10^5^ TcdB-treated B16-F10 cells were injected SC into the right groin of 6-week old C57BL/6 on day −7. A total of 4×10^4^ viable B16-F10 cells, determined by trypan blue exclusion, were inoculated SC into the right flank of the mice on day 0.

The health of mice was monitored daily and tumor size was measured every other day with calipers once the tumors became palpable. Tumor volume was calculated using the formula: length×width^2^×π/6. Differences in mean tumor volume between groups were compared using an unpaired *t-*test. Mice were sacrificed when they bear a tumor in excess of 20∼25% of the body mass or at the end of the observation period by cervical dislocation with standard performance. No mice died before being sacrificed. 30-gauge needles were used for injection to mice, and the injection-volume was never exceeded 100 µl.

### T cell proliferation and IL-2 production

BALB/c mice were immunized twice with 10^6^ TcdB-treated CT26.CL25 cells as described above. Control mice were immunized with saline. Splenocytes from immunized mice were harvested 5 days after the second immunization and cocultured with ovalbumin, recombinant β-galactosidase (10 µg/ml; EMD biosciences, San Diego, CA, USA), or CT26 or CT26.CL25 lysates generated by freeze/thaw cycles. The ratio of splenocytes to tumor cells was 100 to 1. After a 72 h culture, the supernatant from each group was collected, and IL-2 concentrations in the supernatants were determined by ELISA using an IL-2 ELISA kit (Biosource, Camarillo, CA, USA) following the manufacturer’s instructions. For the T-cell proliferation assay, splenocytes were co-cultured with ovalbumin, recombinant β-galactosidase (10 µg/ml), or CT26 or CT26.CL25 lysates for 4 days before the addition of BrdU (EMD biosciences). The cells were harvested 18 h later, and cell proliferation was assayed using the BrdU Cell Proliferation Assay kit (EMD biosciences) following the manufacturer’s instructions.

### Cytotoxic T lymphocyte (CTL) Assay

BALB/c mice were immunized twice with 10^6^ TcdB-treated CT26.CL25 cells. Control mice were immunized with saline. Splenocytes from the immunized mice were harvested 5 days after the second immunization and then cocultured with CT26.CL25 lysate for 5 days. Stimulated effector cells were tested for cytolytic activity against CT26.CL25 cells, parental CT26 cells, or myeloma p3x63Ag8.653 cells using a cytotoxicity detection kit (LDH) (Roche Applied Science, Indianapolis, IN, USA) following the manufacturer’s instructions.

### Statistical analysis

Results are expressed as mean ± standard error of the mean (SEM) unless otherwise indicated. Statistical analysis was performed using Kaplan–Meier survival analysis and by two-tailed *t-*test or one-way ANOVA using the Prism statistical software program (GraphPad Software, Inc., San Diego, CA, USA).

## Results

### Cytotoxic activity of TcdB to CT26 cells

**Figure 1:**
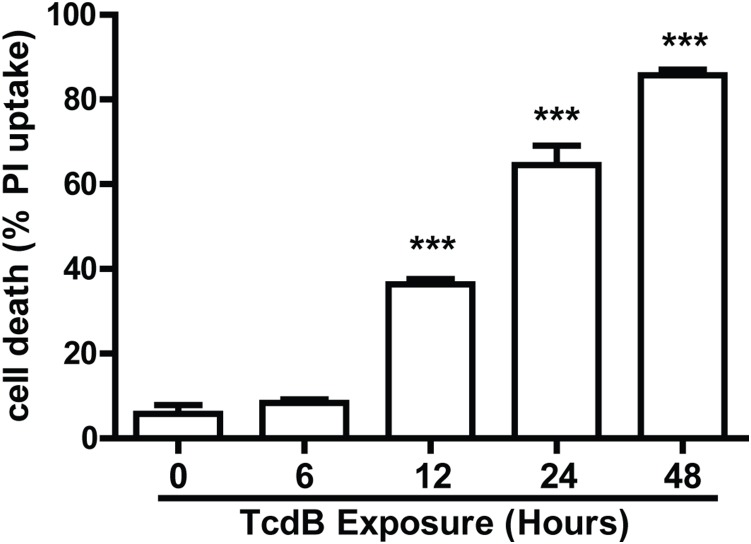
Cell death of TcdB-intoxicated CT26 cells. CT26 cells were exposed to 500 ng/ml of TcdB for different time, and cell viability was measured by the PI staining as described in Material and Methods. Propidium iodide-positive cells were analyzed by Flow cytometry. The data shown represent one of three independent experiments. ***represents P<0.001 vs. control (unpaired two-tailed *t-*test). Error bars, SEM.

The susceptibility of CT26 cells to TcdB-induced cytotoxicity was examined. We determined that 500 ng/ml of TcdB is potent to induce death of CT26 cells by PI staining (Figure 1). The number of PI positive cells was increased after 12 hr of toxin exposure and most of cells became PI-positive within 48 hr of toxin incubation. Six hours of TcdB (500 ng/ml) exposure of CT26 cells did not result in necrotic death of the cells or loss cell membrane integrity, since the PI-positive cells was not increased compared with the control (p = 0.3128; Figure 1). However, after 6 hr of TcdB exposure, all CT26 cells eventually died and no survival cells were observed after 14 days of culture of the intoxicated cells in fresh medium.

### Immunostimulatory effects of TcdB-treated CT26 cells *in vitro*

**Figure 2:**
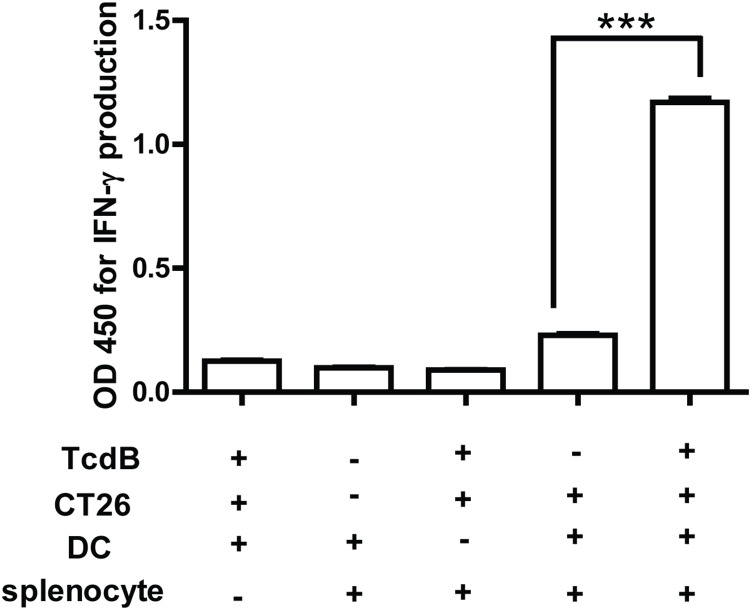
IFN-γ production induced by BMDCs loaded with TcdB-treated tumor cells. Autologous splenocytes were co-cultured with bone marrow DCs (BMDCs) preloaded with live or TcdB-intoxicated CT26 cells for two weeks, and the supernatant was collected to measure IFN-γ production by ELISA. Splenocytes incubated with mere DCs or mere TcdB-treated tumor cells were set as controls. The data represent the mean of three independent determinations ± SEM. ***represents P<0.001 (unpaired two-tailed *t-*test).

Since TcdB is proinflammatory and able to induce intestinal epithelial cells to release cytokines/chemokines [17], [32], we examined the immunostimulatory effects of TcdB-treated tumor cells *in vitro* by testing the ability of DCs loaded with TcdB-intoxicated CT26 cells to activate autologous T cells. BMDCs exposed to TcdB-intoxicated, but not untreated, CT26 cells significantly enhanced IFN-γ secretion (Figure 2). The IFN-γ was produced by T cells but not BMDCs, since the tumor-exposed BMDCs alone did not produce a detectable amount of IFN-γ (Figure 2). In addition, TcdB-treated CT26 cells did not elicit IFN-γ secretion by T cells in the absence of DCs (Figure 2), indicating that the intoxicated CT26 cells could not directly induce T cell production of IFN-γ but rather via activation of DCs for subsequently T cell activation. BMDCs matured by LPS failed to induce T cell production of IFN-γ (Figure 2), suggesting that tumor-specific response is required for the IFN-γ secretion. Taking together, these data demonstrate that TcdB-intoxicated CT26 cells have the potent capacity to stimulate the activation of BMDCs and subsequent T cell activation.

### Induction of anti-tumor immunity *in vivo*

**Figure 3:**
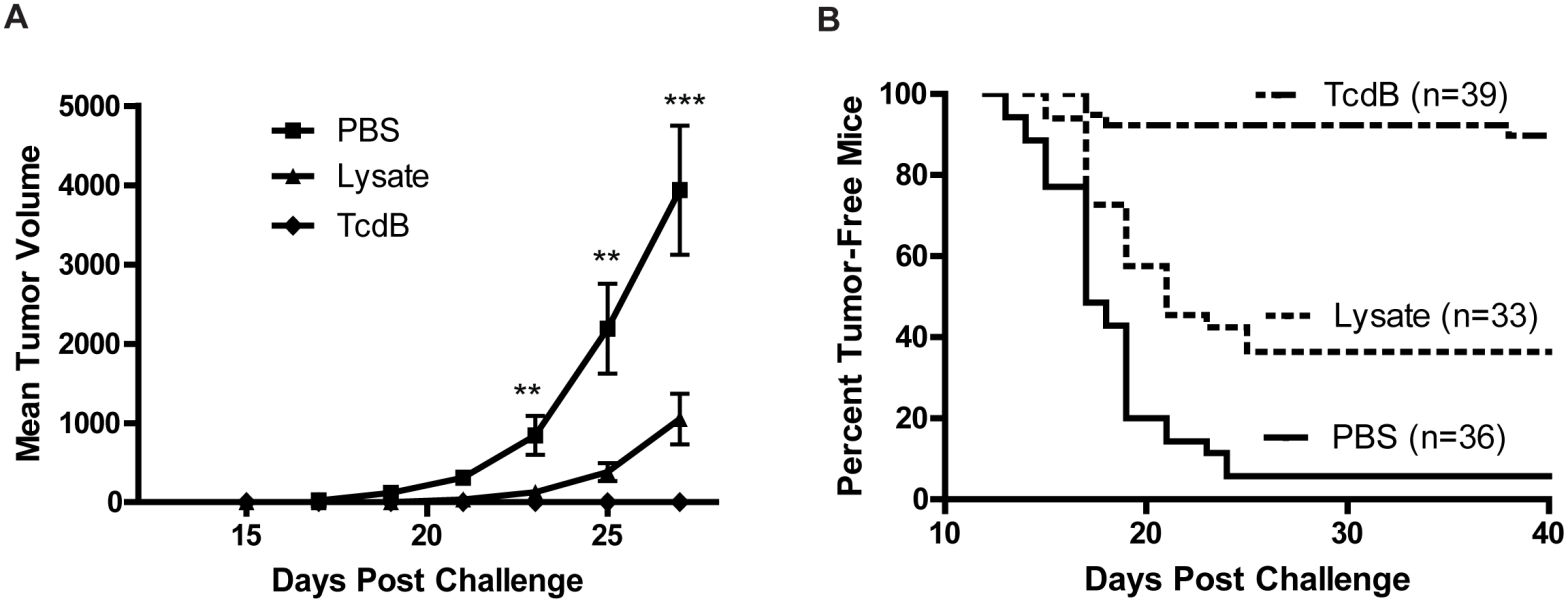
Anti-tumor immunity induced by dying CT26 cells. Mice were subcutaneously immunized with PBS or TcdB-exposed CT26 cells (TcdB). In some experiments, mice were injected with lysate of TcdB-treated CT26 cells (TcdB-lysate). Mice were then challenged with 10^5^ live CT26 cells on the opposite side of the groin and tumor growth was monitored. (A) Tumor volume was calculated using the formula: length×width^2^×π/6. The data represent one of five independent experiments (n = 5∼8). **, P<0.01 vs. PBS; ***, P<0.001 vs. PBS (paired two-tailed *t-*test). Error bars, SEM. (B) The percentage of tumor-free mice was measured. The data in (B) represent a pool from five independent experiments (n = 5∼8 for each experiment).

The immunostimulatory activity of TcdB-intoxicated CT26 cells prompted us to investigate the ability of the dying cells for inducing antitumor immunity *in vivo*. Vaccination of mice with TcdB-treated CT26 tumor cells induced potent anti-tumor immunity. Mice immunized with TcdB-intoxicated cells rejected a lethal dose of CT26 challenge, whereas PBS-immunized mice developed tumors rapidly (Figure 3A). The pooled data (Figure 3B) from five independent experiments showed that only 4 out of 39 mice (10%) immunized with TcdB-intoxicated CT26 cells developed tumors, whereas 94% grew tumors after vehicle (PBS) immunization (Figure 3B, p<0.0001).

### Role of cell integrity in the induction of anti-tumor immunity

Since the intoxicated cell maintained their membrane integrity and were PI-negative before injection, we sought to determine whether the cell membrane integrity of tumor cells is important for their ability to induce antitumor immunity. TcdB-intoxicated CT26 cells were freeze-thawed for 5 cycles before immunizations. Repeated freeze-thaw treatment significantly decreased the immunogenicity of the intoxicated CT26 cells (Figure 3A, B). Although mice that were immunized with tumor cell lysate exhibited retarded tumor growth and a reduced frequency in tumor-bearing mice as compared to the PBS group, the potency of the anti-tumor immunity was significantly reduced in comparison to intoxicated-tumor cells without lysis (Figure 3A, B). Over 50% of mice that were immunized with CT26 cell lysate grew tumors, which was substantially higher than mice immunized with intact TcdB-exposed tumor cells in which less than 10% of mice developed tumors (Figure 3B, p<0.0001). These data indicate that intact cell bodies are critical for the potent immunogenicity of TcdB-treated CT26 cells.

### Induction of type I cytokines and specific cytotoxic T lymphocytes (CTLs)

**Figure 4:**
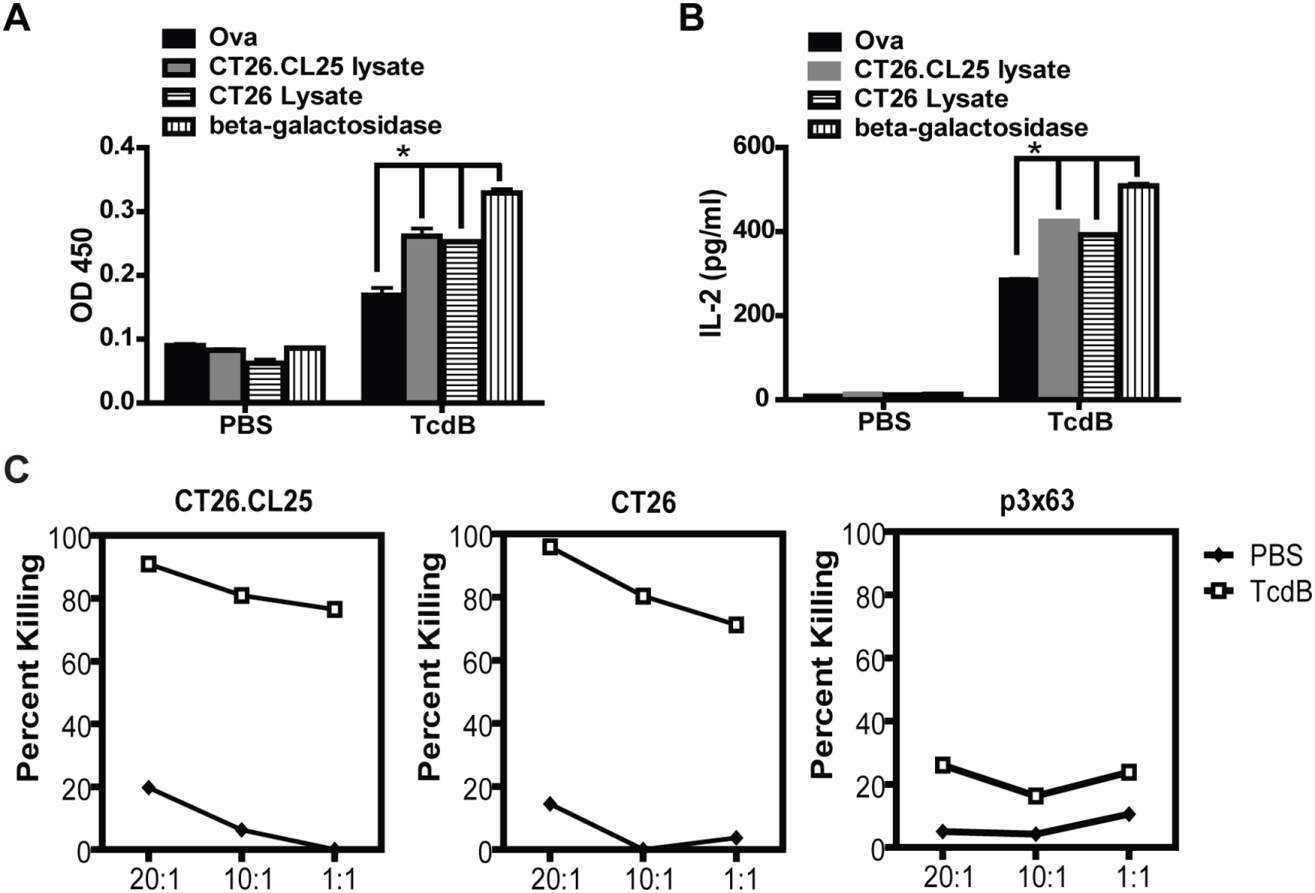
T-cell proliferation, IL-2 secretion, and specific CTL activity of splenocytes from immunized mice. Mice were immunized twice with PBS or TcdB-intoxicated CT26.CL25 (TcdB), and splenocytes were harvested 5 days after the second immunization. (A and B) splenocytes were restimulated with OVA, CT26.CL25 lysate, CT26 lysate, and β-galactosidase. (A) T-cell proliferation was determined by BrdU cell proliferation assay. (B) IL-2 production was measured by ELISA. The data in (A) and (B) represent the mean of three independent experiments ± SEM. *represents P<0.05 (one-way ANOVA). (C) Specific CTL induction. Splenocytes restimulated with CT26.CL25 lysate were tested for cytolytic activity against CT26.CL25 cells, parental CT26 cells, or myeloma p3x63Ag8.653 cells using cytotoxicity detection kit (LDH) assay. Representative data from one of three independent experiments are shown.

Cell-mediated immunity plays an essential role in combating tumors [33] and is characterized by the production of type I cytokines, such as IL-2 and tumor necrosis factor α (TNF-α), and the induction of CTLs. To explore whether vaccination with TcdB-treated CT26 cells could induce type I cytokine secretion by T cells and generate tumor-specific CTLs, we examined IL-2 production, T cell proliferation, and cytolytic activities of splenocytes derived from vaccinated mice. When mice were immunized twice with TcdB-intoxicated CT26 cells expressing a model antigen, β-galactosidase (CT26.CL25 cells) [34], their splenocytes proliferated more vigorously in response to *in vitro* stimulation with either CT26.CL25, its parent CT26 cell lysate, or purified recombinant β-galactosidase antigen, rather than irrelevant antigen ovalbumin (Figure 4A). The moderate proliferation of splenocytes from mice immunized with TcdB-treated CT26.CL25 cells was detected when incubated with ovalbumin *in vitro* (Figure 4A). This may be because that some splenocytes remained active 5 days post the second immunization with TcdB-treated tumor cells. Similarly, splenocytes secreted more IL-2 in response to tumor lysates or β-galactosidase than in response to ovalbumin (Figure 4B). T cell proliferation and IL-2 production stimulated by the tumor lysates or the purified recombinant protein were specific since splenocytes from mice given a placebo (PBS) immunization failed to respond to these stimuli (Figure 4A, B).

We further examined the CTL activity of splenocytes from the vaccinated mice. Splenocytes from immunized mice were restimulated with CT26.CL25 lysate for 5 days and then assessed for cytolytic function against different tumor targets. Vaccination with TcdB-intoxicated tumor cells elicited potent and specific CTL activity against either CT26.CT25 or its parental cell line CT26 but not the irrelevant autologous tumor cell line p3x63Ag8.653 (p3x63) (Figure 4C). Specific CTL activity of splenocytes may suggest that the main T cell response elicited by the immunization with intoxicated CT26.CL25 cells is tumor specific.

### Protection against pre-injected tumors

**Figure 5:**
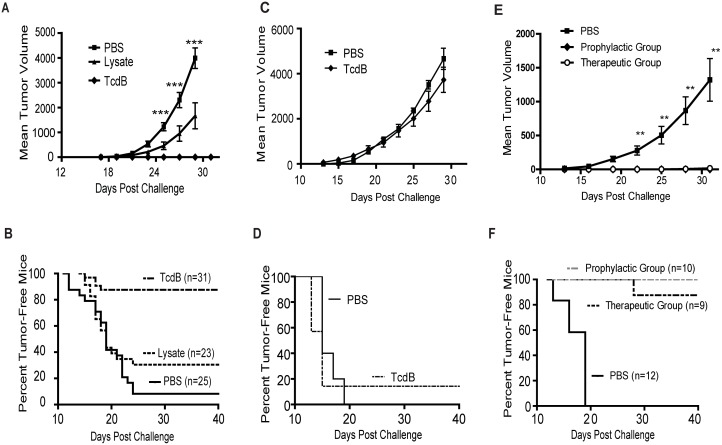
Specific and long-lasting anti-tumor immunity induced by TcdB-treated CT26 cells. (A and B) Protection against pre-injected tumors. Four hours after lethal CT26 tumor cell challenge, mice were injected with PBS, TcdB-exposed CT26 cells (TcdB), or CT26 lysate (TcdB-lysate). (A) Tumor volume was measured. The data represent one of four independent experiments (n = 5∼8). ***represents P<0.0001 vs. PBS (paired two-tailed *t-*test). Error bars, SEM. (B) The percentage of tumor-free mice was determined. The data presented is a pool from four independent experiments (n = 5∼8 for each experiment). (C and D) Mice vaccinated with TcdB intoxicated CT26 cells are not protected against myeloma p3x63 cells. Mice were challenged with lethal myeloma p3x63 cells and then immunized with TcdB-intoxicated CT26 cells (TcdB) or vehicle control (PBS) at a different site 4 h later. (C) Mouse tumor volume was measured (n = 8). (D) The percentage of tumor-free mice was measured (n = 8). (E and F) The anti-tumor immunity induced by TcdB-intoxicated tumor cells is long lasting. Mice surviving the first challenge with CT26 cells after either prophylactic or therapeutic vaccination with TcdB-treated tumor cells were rechallenged with 10^6^ (10 times the LD100) CT26 cells 3 months after the first challenge. The age-matched naive mice were challenged with 10^6^ CT26 cells as control. Tumor volume (E) and the percentage of tumor-free mice (F) were evaluated. The data shown represent one of three independent experiments. ** in e, P<0.01 between prophylactic group or therapeutic group vs. PBS (paired two-tailed *t-*test); *** in f, P<0.0001 vs. PBS (Log-rank test). Error bars, SEM.

We further assessed the potency of anti-tumor immunity mediated by vaccination with the intoxicated CT26 cells. Mice were given a single immunization 4 h after the transplantation of CT26 cells at a different site. In the PBS-immunized mice tumors grew rapidly (Figure 5A) with most mice (>92%) developing tumors within 25 days post-challenge (Figure 5B), whereas only 4 of 31 mice that were vaccinated with TcdB-intoxicated CT26 cells developed tumors (Figure 5B, p<0.0001). Disrupting the membrane integrity of tumor cells by freeze/thawing (lysate) significantly decreased the immunogenicity of TcdB-intoxicated tumor cells, resulting in a significantly reduced ability to retard tumor growth and reject pre-injected tumors in these vaccinated mice (Figure 5A, B). Only 30% of vaccinated mice in lysate group were completely tumor-free, compared to more than 87% of mice that never grew tumors in the group vaccinated with intact intoxicated tumor cells (Figure 5B, p<0.0001).

### Specificity and longevity of anti-tumor immunity

We investigated whether the *in vivo* anti-tumor immunity induced by TcdB-intoxicated tumor cells is specific. Mice immunized with TcdB-intoxicated CT26 cells grew p3x63 tumors similarly to PBS-immunized mice (Figure 5C, D) but were protected against challenge with CT26 cells (Figure 5A, B), suggesting that the anti-tumor immunity was indeed tumor-specific. Furthermore, this anti-tumor immunity was long-lasting. Both age-matched naïve mice and surviving mice from those immunized either prophylactically (prophylactic group) or post-initial challenge (therapeutic group) were rechallenged with 10×LD_100_ of CT26 tumor cells 3 months after the initial challenge. The age-matched naïve group grew tumors rapidly whereas none of the mice from the prophylactic group developed any tumors over the 40 days of the observation period (Figure 5E, F). One out of nine mice from the therapeutic group developed tumors 28 days post rechallenge whereas the rest of the mice in this group remain tumor-free until the end of the experiment (Figure 5F).

### Induction of anti-tumor immunity in a melanoma model

**Figure 6:**
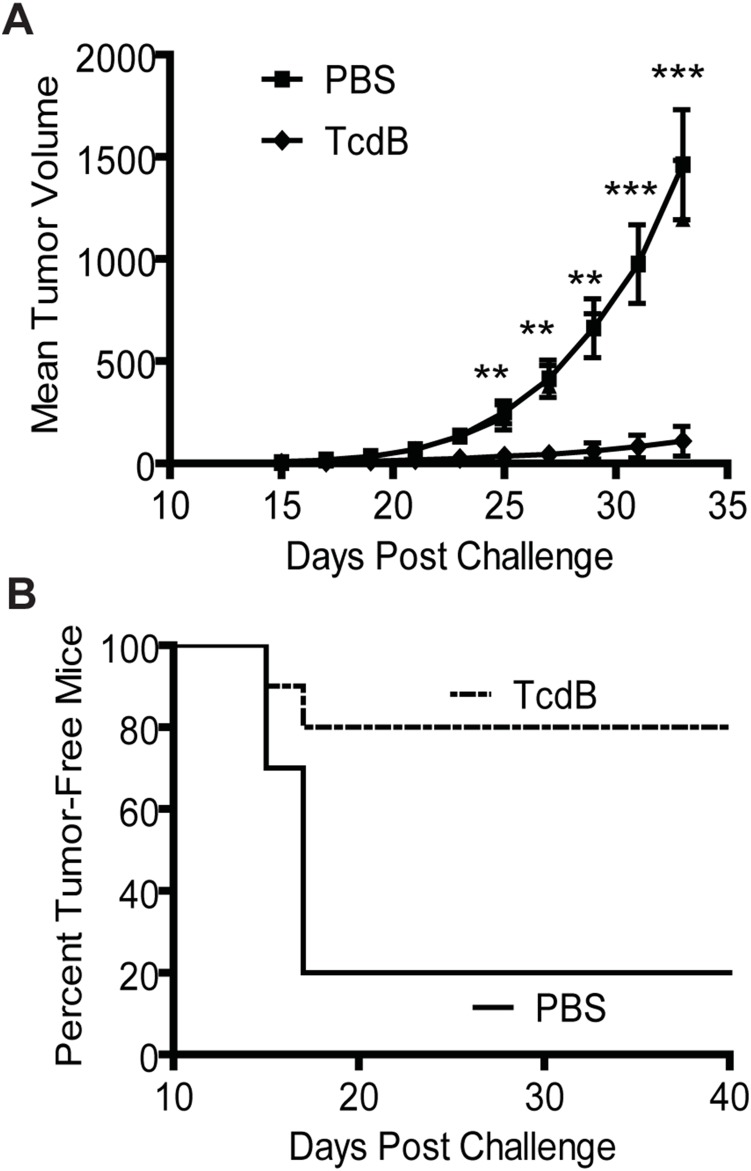
Induction of an anti-tumor immune response by TcdB-intoxicated B16-F10 cells. Mice were immunized once with PBS or 2×10^5^ TcdB-exposed B16-F10 cells per mouse before challenge with lethal B16-F10 cells. Tumor volume (A) and the percentage of tumor-free mice (B) were measured. Representative data from one of three experiments are shown (n = 10 for each experiment). **, P<0.01 vs. PBS; ***, P<0.001 vs. PBS (paired two-tailed *t-*test). Error bars, SEM.

Finally, we investigated whether the potent immunogenic tumor cell death induced by TcdB was limited to the colorectal tumor CT26 in Balb/C mice. To examine this, we utilized a well-studied mouse melanoma cancer (B16-F10) model in C57BL/6 mice. Consistent with the findings in the CT26 model, vaccination of mice with TcdB-treated B16-F10 cells induced significant protection against a lethal challenge of B16-F10 tumor cells. Tumor growth was substantially inhibited compared to control mice immunized with vehicle PBS (Figure 6A). Only 2 of 10 mice (20%) immunized with TcdB-intoxicated B16 cells developed tumors, whereas 8 of 10 mice (80%) developed tumors in the PBS group (Figure 6B, P<0.05). Thus, TcdB can be used for developing anti-tumor immunity against multiple types of cancers.

## Discussion

*Clostridium difficile* is a major health care concern causing serious and potentially fatal through two major toxins, TcdA and TcdB [35], [36], [37]. Although both toxins are cytotoxic to cultured cells, TcdB is generally 1000-fold more potent than TcdA, capable of killing target cells in femtomolar dose ranges [10]. In this study, we demonstrated that tumor cells intoxicated by TcdB are highly immunogenic, capable of activating DCs and stimulated potent and long-lasting antitumor immunity in mice. Our results thus provide new insight for utilizing *C. difficile* exotoxins for inducing antitumor responses.

*C. difficile* toxins induce cell death through apoptosis or necrosis [11], [12], [13], [14], [15], which may depend upon the dose of toxins and cell types. We have previously showed that mouse colorectal adenocarcinoma CT26 cells are highly sensitive to TcdB induced cell death [38]. In this study, we found CT26 cells maintained their cell membrane integrity during the 6 hr of TcdB (500 ng/ml) exposure, suggesting that TcdB did not induce early necrotic death in these cells. Nevertheless, all intoxicated CT26 cells eventually died after cultured in fresh medium, indicating that there were enough glucosyltransferase toxin being internalized and caused irreversible damages in host cells. The reduction in immunogenicity by lysing the toxin-exposed CT26 indicates that the anti-tumor immunity was elicited by the intoxicated cells, rather than by TcdB associated with the cells, since tumor lysates were exposed to the same amount of the toxin.

The underlying mechanism for the intoxicated tumor cells to induce antitumor immunity is unclear, but the effector function of the glucosyltransferase activity of the toxin is likely required. We have previously demonstrated that the effector glucosyltransferase activity of *C. difficile* toxins is required for the induction of proinflammatory cytokine TNF-α by macrophages [39]. A mutant TcdB deficient with glucosyltransferase activity is unable to induce death of CT26 cells even at 1000 ng/nl for 72 hrs [40]. In this study, we found that disruption of membrane integrity of the intoxicated tumor cells significantly reduced their ability to induce antitumor immunity, indicating that intact tumor cells may be necessary for producing immunostimulatory molecules that are important for the induction of antitumor immunity. It has been reported both *C. difficile* toxins are proinflammatory and capable of inducing cytokines/chemokines in host cells [5], [16], [17]. The proinflammatory cytokines likely contribute to the activation of DCs and subsequently stimulate potent antitumor immunity. However, further studies are necessary to elucidate the possible mechanisms.

The induction of potent immunogenic death in tumor cells by TcdB has implications in designing anti-tumor vaccines. Early studies evaluated the direct antitumor effects of the toxins on cultured tumor cells and *in vivo* tumor growth in nude mice [8], [9], [41]. However, few studies to combine the tumor killing of bacterial toxins with their ability to induce antitumor immunity [30], [31]. TcdB is highly toxic to a broad range of cell types [10], and the induction of immunogenic cell death by the toxins occurs in different tumor models. Therefore, TcdB may be used in generating vaccines against a wide variety of tumors. The utilization of immunogenic properties of cancer cell death has been considered as an ideal strategy to improve the outcome of cancer therapy [22], [23], [42], and previous study showed that some chemical drugs have an ability to induce immunogenic death of tumor cells [27], [28]. Compared with chemical reagents, bacterial toxins can be engineered to specifically target tumor cells. Thus our study may provide insight into designing novel immunotoxins based on *C. difficile* toxins, allowing targeted killing of tumor cells as well as inducing specific anti-tumor immunity *in vivo*.
